# The PharmNet Harm Reduction Intervention for Community Pharmacies: Protocol for a Pilot Randomized Controlled Trial

**DOI:** 10.2196/42373

**Published:** 2022-10-24

**Authors:** Lori Ann Eldridge, Jon Agley, Beth E Meyerson, Lilian Golzarri-Arroyo

**Affiliations:** 1 East Carolina University Greenville, NC United States; 2 Prevention Insights, Department of Applied Health Science School of Public Health Bloomington Indiana University Bloomington Bloomington, IN United States; 3 Harm Reduction Research Lab, Southwest Institute for Research on Women College of Social & Behavioral Sciences University of Arizona Tucson, AZ United States; 4 Family and Community Medicine College of Medicine University of Arizona Tucson, AZ United States; 5 Biostatistics Consulting Center School of Public Health Bloomington Indiana University Bloomington Bloomington, IN United States

**Keywords:** naloxone, opioid, overdose, pharmacy, randomized controlled trial, RCT, opioid use, digital health intervention, community health, drug use, prevention, PharmNet, health resources, health outcome

## Abstract

**Background:**

The overdose epidemic in the United States has continued to worsen despite substantial efforts to mitigate its harms. The opioid antagonist naloxone has been identified as a key means of reducing the prevalence of fatal overdoses. An important evidence-based approach to optimizing naloxone’s impact is to seed it throughout the community, because bystanders are often able to reverse overdoses more quickly than first responders and sometimes are the only possible means of overdose reversal. As part of a multipronged approach to distributing naloxone nationwide, community pharmacies have been identified as ideal venues for naloxone dispensing, especially under standing orders. However, dispensing rates remain surprisingly low, and there is a need to understand how best to engage community pharmacies in naloxone-based harm reduction services.

**Objective:**

The objective of this trial is to determine whether a tailored, pragmatic pharmacy intervention (PharmNet) results in greater naloxone dispensing relative to baseline (the prior 3 months) compared to a control condition. This pilot trial is intended to determine whether it is appropriate to invest the substantial resources that would be required to conduct a full-scale, randomized controlled study of PharmNet.

**Methods:**

We will conduct a 3-month randomized controlled pilot trial consisting of 2 parallel groups with a 4:3 allocation ratio. A group of 7 independent pharmacies from rural areas in Indiana will be randomly assigned to either the PharmNet intervention arm (n=4) or the control arm (n=3). The primary outcome will be overall naloxone dispensing (both at cost and free), and secondary outcomes will include the distribution of referral cards and multiple variables at the level of individual staff members. Dispensing data will be collected for the 3 months prior to the intervention and the 3 months of the intervention, and all other data will be collected using a pretest-posttest design. The primary analysis will be a generalized linear mixed model with a Poisson distribution with fixed effects for group, time, and their interaction and a random effect for pharmacy ID to account for repeated measures within pharmacies.

**Results:**

This study was approved by the Indiana University institutional review board in 2 phases (August 2, 2021, and April 26, 2022) and was funded by the Indiana University Grand Challenge: Responding to the Addictions Crisis.

**Conclusions:**

If this study produces evidence that the PharmNet intervention results in increased naloxone dispensing relative to control pharmacies, it will be both appropriate and important to study it in a large, full-scale randomized controlled trial.

**International Registered Report Identifier (IRRID):**

PRR1-10.2196/42373

## Introduction

### Overdose Deaths in the United States

The prevalence of overdose deaths in the United States has continued to increase in recent years, eclipsing 100,000 such deaths (year over year) for the first time in June 2021 [1]. The majority of such deaths involve opioids; more specifically, in the current wave of the overdose epidemic, they involve heroin, fentanyl, and its analogues—the latter 2 of which are often introduced as adulterants in other substances (eg, stimulants) [1,2]. Largely related to such deaths, the United States renewed a declaration of an opioid public health emergency in April 2022 [3]. In the state of Indiana, where the proposed study will take place, provisional data indicated 2554 fatal drug overdoses in 2021, of which more than 78% involved opioids [4].

Naloxone, an opioid antagonist, has become a key component of the US response to the crisis [5], as it can reverse the effects of an overdose and prevent death [6,7]. However, the effectiveness of naloxone depends on the time it is administered relative to the time of the overdose. Deaths from heroin overdose can occur relatively quickly [8], and even when they do not, they typically happen within 1 to 3 hours of overdose [9]. Deaths from fentanyl overdose may occur even more rapidly [10], potentially due to differences in pharmacology between fentanyl and other opioids [11,12]. Unfortunately, individuals who witness an overdose may be hesitant to contact first responders [13,14], and even when they do, there is still variable lag time between the call for assistance and the arrival of assistance due to a variety of factors (eg, distance from the dispatch site to the overdose location). In some cases involving fentanyl, death may occur before first responders arrive [10]. Thus, there is an ongoing need to better ensure that naloxone is readily available to prevent overdose deaths.

In 2018, the US Surgeon General recommended that all people in the United States be prepared by having naloxone on hand to help in case of an overdose [15]. That recommendation followed, and has been followed by, evidence that overdose education and layperson and/or bystander administration of naloxone are safe and effective approaches to reducing the likelihood of opioid overdose fatality [16-19]. Similar to many states, Indiana has adopted laws to facilitate community overdose reduction using naloxone [20], including “good Samaritan” laws [21]. Although the means by which people obtain naloxone and other information related to harm reduction remain diverse, our focus in this proposed study is on community pharmacies.

### Role of Community Pharmacies in Harm Reduction

Community pharmacies are well positioned to provide harm reduction services to all community members, along with more targeted services to individuals at risk of overdose. Almost 9 in 10 Americans live within 5 miles of a community pharmacy [22], meaning pharmacists are among the most accessible health care professionals [23]. Among some populations (eg, Medicare beneficiaries), it has been shown that pharmacists interact with patients more often than their primary care physicians [24].

In addition to their accessibility, pharmacies increasingly are perceived as effective venues for both clinical and nonclinical harm reduction owing to pharmacists’ expertise in fields such as medication management and pharmaceutical education [25,26]. A study of Arizona pharmacists found that even when uncomfortable with most forms of harm reduction, pharmacists were generally comfortable with dispensing naloxone [27]. In Indiana, qualitative data from managing pharmacists found conceptual support for harm reduction but, simultaneously, concerns about systemic barriers to implementation, including a lack of time for interventions as well as concerns about role clarity and patients’ expectations of what should happen in a pharmacy [28]. Similar patterns of support and concern were raised in a more recent study of key informants (including pharmacists) in Connecticut, Kentucky, and Wisconsin, reflecting willingness to discuss naloxone with patients but barriers related to role clarity as well as the financial cost of naloxone [29].

Thus, despite comfort and willingness to support harm reduction and treatment, as well as faciliatory state laws (eg, standing orders permitting naloxone dispensing without a patient-specific prescription), a recent systematic review found that naloxone was stocked on average in 62.8% of community pharmacies, with both the likelihood of stocking and willingness to dispense without a prescription significantly lower in independent pharmacies than in chain pharmacies [30]. That finding was echoed by a contemporary analysis of naloxone nasal spray stocking in 11 US states, which reported a similar rate (69.5%) and a significantly lower likelihood of stocking among independent pharmacies [31].

### Our Approach to Harm Reduction Intervention in Pharmacy: the Proposed Study

The proposed study will be a pilot randomized controlled assessment of the effectiveness of PharmNet, a harm reduction intervention for community pharmacies focused on naloxone distribution, awareness building, and referral. As described in the write-up of our single-site pilot study, “the intention was to study procedures that have as minimal an impact as possible on pharmacy costs and operational functioning while maximally facilitating harm reduction from opioid overdose—in other words, to find an optimal intersection point of those concerns” [32]. In brief, the intervention includes [32]:

- Building awareness of naloxone availability at the site among patients (eg, the use of yard signs and scrolling messages on television screens);
- Supporting awareness among pharmacists and pharmacy technicians about proactively offering naloxone (eg, customized post-it notes);
- Facilitating service provision by conducting a priori negotiations and establishing written agreements with local nonprofits to facilitate a pipeline of no-cost naloxone for the pharmacy;
- Emphasizing bidirectional naloxone provision (eg, pharmacist- and pharmacy technician–initiated offers in addition to patient-initiated requests); and
- Facilitating referral by providing a physical, durable, and curated list of community resources that can be used by pharmacists, handed to patients, and placed into pharmaceutical bags.

Our previous single-site pilot study found that during the intervention, compared to the mean of the 3 months of practice prior to the intervention, the monthly rate of naloxone sold had a relative increase of 34.4% (+3.33 doses/month), and the overall naloxone dispensing rate through *any* mechanism (eg, including newly added no-cost dispensing) increased 96.48% (+9.33 doses/month) [32].

Although the intervention is conceptually simple, the decisions made in its development involved highly intentional design. Changes to the program were rooted in the Consolidated Framework for Implementation Research because it allows for considerations to be addressed within complex settings, such as pharmacies [33]. The PharmNet team conducted numerous information gathering, feasibility, and methodological studies in preparation for the single-site pilot to obtain increasingly specific information and context, including both quantitative and qualitative work. Much of this work was conducted in conjunction with academic and community-based pharmacists and involved data collection from hundreds of managing pharmacists in 2 states [27,28,34-42]. The single-site pilot itself was informed by independent community pharmacists at a more granular level to maximize likely acceptability and feasibility [32].

### Study Objectives and Hypotheses

Our study will be designed to facilitate an improved understanding of whether it is reasonable to believe that the PharmNet intervention causes increased dispensing of naloxone by randomizing independent pharmacies that are part of the same small pharmacy chain to either (1) the PharmNet intervention or (2) operations as usual (control). It will also explore multiple secondary outcomes and questions.

- Hypothesis 1: Monthly naloxone dispensing (combined sales and no-cost distribution) will be significantly increased in the pharmacies implementing PharmNet compared to those in the control arm.
- Other preregistered analyses: This study will observe the volume of PharmNet referral cards distributed by the intervention sites. It will also collect several measures from staff members (pharmacists and technicians) to provide a broader understanding of the intervention, such as staff members’ comfort with multiple harm reduction practices, stigmatizing beliefs about people who use drugs, and perceptions of intervention acceptability.

## Methods

### Ethics Approval

This study will comply with all ethical regulations as outlined and approved by the Indiana University institutional review board (IRB; 12339 and 13956). The pharmacy employee data collection for this study was approved with a designation of “exempt” from the Indiana University IRB (13956).

### Consent to Participate

Pharmacy employees who choose to participate in the staff-specific components of the study will complete an electronic consent form prior to providing any data. The completion of the pretest survey will be compensated by a US $5 digital gift card, and the completion of the posttest survey will be remunerated by a US $10 digital gift card. All other components of PharmNet have been deemed an organizational level research project and received a not human subjects research designation from the Indiana University IRB (12339). Participating pharmacy sites will each be paid US $1000 following the successful completion of this pilot study based on estimated cost data provided to author JA by the parent organization following the single-site pilot study.

### Confidentiality

Pharmacy employees’ responses will be linked to their provided email addresses in a survey platform (QualtricsXM) that is approved by Indiana University for “critical data,” the highest security designation. Survey responses will not be shared outside of the research team, and data will only be reported and analyzed in aggregate.

No other identifiable data will be obtained by the research team (eg, naloxone-dispensing data will be obtained only in aggregate for the prespecified time periods).

### Design

#### Trial Design

**Figure 1:**
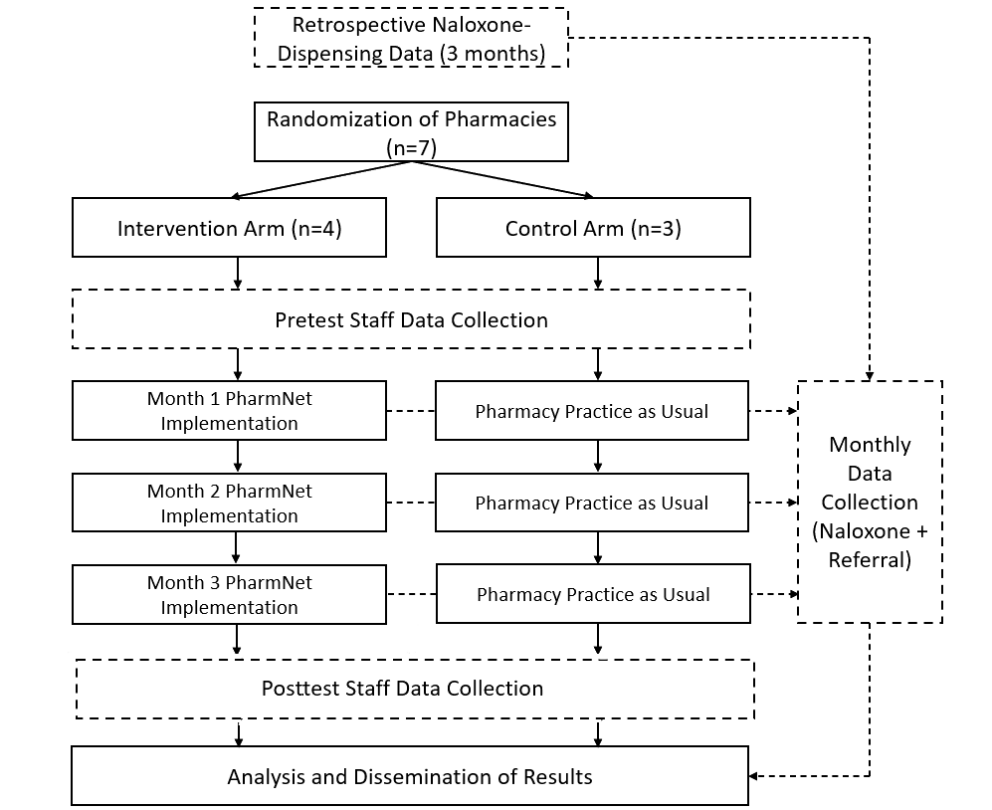
Project flow diagram.

The PharmNet pilot trial will be a cluster randomized trial consisting of 2 parallel groups and a 4:3 allocation ratio of intervention to control. The independent pharmacy organization participating in the project consists of 8 pharmacies, of which 1 was the pilot and feasibility test site [32]. The remaining 7 pharmacies will be allocated using a web-based random sequence generator (conducted by author LAE), such that 4 pharmacies will be selected to implement PharmNet, and 3 pharmacies will serve as controls (pharmacy practice as normal). The workflow for the study is provided in Figure 1**.**

This study will utilize partial blinding; the research team and the pharmacy manager (who supervises all 7 pharmacies, see description in our prior paper [32]) will not be blinded to assignment because it would not be possible to do so, but individual pharmacists, pharmacy technicians, and others working in the control arms will not be aware that their pharmacies are serving as controls.

#### Participants and Procedures

This study will be conducted with an Indiana family-owned independent pharmacy system consisting of 8 pharmacies. The organization has been operational for over 120 years and predominantly operates in rural communities. Its pharmacies offer a variety of services including infusions, durable medical equipment, medication delivery, and cost containment. This organization reports continually seeking ways to expand its services to meet community needs.

The pharmacy manager has already agreed in principle that the organization sites will participate in this pilot cluster randomized trial. Data collected at the site level will be obtained from these entities, which are the only eligible pharmacies (n=7). Within each pharmacy, all pharmacists and pharmacy technicians will be asked to complete 2 questionnaires. These individuals will be asked to provide individual-level consent and will not be required to complete the questionnaires.

Data from individual staff members will be collected prior to the intervention (a 2-week window before the start date) and after the intervention (a 2-week window after the end date). Staff members who are hired after the intervention begins will be eligible to take the second survey (posttest) but not the first (pretest). Aggregated data (ie, counts) on naloxone dispensing from the pharmacies will be collected retrospectively (the 3 months prior to the intervention initiation) and then monthly for the 3-month duration of the intervention using the pharmacies’ record systems. Data on the distribution of additional items (no-cost naloxone and referral cards) will be obtained through staff member counts of products at predetermined monthly intervals (all items will be numbered to facilitate accuracy).

#### Intervention Description

The development of this intervention and the decisions made during that process, including the recent changes that were informed by the single-site pilot study, are described in detail in a prior paper [32]. Therefore, we have prepared a structured table (see Table 1) outlining the sequential steps in the intervention and refer readers to our prior paper for additional detail.

**Table 1:** Intervention components.

Intervention stage	Steps	Other notes
Introduction to study (prior to study start date)	Digital video (.mp4) describing the intervention (~5 minutes in length) is distributed to the pharmacy manager. Pharmacy manager disseminates the video. All staff members view the video. Lead pharmacists are asked to include the video in the orientation package for new pharmacists and pharmacy technicians and those who may have missed the initial meeting.	Each pharmacy has a regular, site-specific “all hands” meeting each month. In the pilot and feasibility study, this meeting was identified as the appropriate venue to disseminate intervention information.
Early implementation (prior to study start date)	Confirm with the managing pharmacist that all intervention pharmacies have viewed the video at the “all hands” staff meeting. Deliver a set of 30 doses of naloxone (presently, individually packaged Narcan nasal spray) to each pharmacy in numerically marked bags imprinted with “Not for Sale.” Deliver a set of 500 consecutively numbered harm reduction referral slips to each pharmacy (customized for each pharmacy location). Deliver sets of reminder post-it notes to pharmacies. Deliver the requested number of yard signs to each pharmacy (based on the number of routes of travel around the pharmacy). Ensure that pharmacy’s in-store television has access to the scrolling reminder text.	Digital copies of all of the visual materials described here (3, 4, and 5) are freely available in a prior publication [32].
Launch (on the study start date)	Reminder post-its placed in visible areas on the interior of the dispensing location (eg, staff side rather than patient side). Harm reduction referral cards placed at each checkout for easy access. Yard signs placed outside the building in visible locations where traffic (vehicle or foot) is common. Scrolling television banner will be enabled to run continuously through the intervention. “76.7% of overdoses happen at home. Bystanders who witness an overdose can be effective in reducing overdose mortality. Ask us about how you can save a life with naloxone today!”	Digital copies of all materials for this project are provided as attachments to the single-site pilot study manuscript [32]. All marketing materials will be standardized, except that the referral and recovery resource information (referral cards) will be specific to each local community.
Implementation (continuous after start)	There are 4 primary procedural events that result in offers of naloxone as well as provision of harm reduction cards. Three are initiated on the pharmacy side and were tailored for this project based on harm reduction management plan recommendations for clinicians from the Centers for Disease Control and Prevention [43]. Those are: 1. Prescription fill for a medication for opioid use disorder; 2. High-dose opioid prescription (50 morphine milligram equivalents or greater); 3. Request for syringes without a prescription, even if syringes are not available at the site. The fourth is initiated on the patient side: 4. Patient request for naloxone. The existence of these guidelines is specifically not intended to preclude offers or dispensing in other situations (eg, pharmacist discretion).	Naloxone will be dispensed based on the patient’s ability to pay, as determined by the pharmacist or pharmacy technician. Referral cards will be provided at each procedural event regardless of whether naloxone is dispensed. Pharmacy staff may either briefly acknowledge the card (“We wanted to share with you some community resources that may interest you”) or not. The cards can be placed inside the prescription bag (they are one-quarter of a standard 8.5 × 11 in page) or handed directly to the patient.
Ongoing supplies (continuous after start)	The pharmacy manager, as well as the lead pharmacist at each site, will be instructed to contact the research team a minimum of 3 business days prior to any intervention component (eg, naloxone, referral cards, and reminder post-its) being exhausted. The research team will then provide additional supplies.	Based on the single-site pilot, the initial materials provided at launch are expected to be sufficient for the project duration.
Fidelity monitoring (~1 month after start)	Approximately 1 month after the intervention’s launch, we will conduct a single fidelity site visit to each intervention site. This will include inspection for presence of all materials (eg, yard signs, television messaging, and field observations). Any major concerns will be addressed with the pharmacy manager.	Since our orientation to this project is pragmatic, we do not plan to engage in extensive fidelity monitoring, as this would not be available in a real-world implementation of the program [44].

An essential component of the PharmNet intervention is that pharmacies have naloxone on hand that they can dispense at no cost to patients who cannot afford it. Such dispensing accounted for roughly two-thirds of the overall increase in dispensing in our single-site pilot [32]. However, we had identified that the independent pharmacies in this project did not have direct access to free or reduced cost naloxone and often must order it in response to prescriptions. Thus, as part of the intervention, the study team developed memoranda of understanding (MOUs) with (1) one of the largest nonprofit agencies that provides free naloxone to the state of Indiana, (2) a secondary nonprofit as a “backup,” and (3) the pharmacy organization. These MOUs were initially designed to serve for the duration of the project and facilitated the structured transfer of naloxone at the needed volume to participating pharmacies at no cost. The MOUs did not reference a specific limit on available doses but indicated the possibility that the nonprofit may, at times, not have free naloxone available to distribute—leading to our engagement of the “backup” organization. Importantly, now that procedures and agreements are in place, the study team will be able to “withdraw” from the MOUs at the end of the study and facilitate similar MOUs directly between the nonprofits and pharmacy organization to continue the supply of naloxone without an intermediary. Facilitating this supply of naloxone to the pharmacy was perceived as critical to the program’s success in the single-site pilot [32] and may be important to supporting the sustainability of ongoing programs of this kind.

### Sampling Plan: Sample Size Determination and Power Analysis

As noted above, we will conduct this study among 7 independent community pharmacies, with a 4:3 allocation to intervention and control arms. With 80% power (α=.05, 2-tailed), this sample will allow us to detect large effect sizes (Cohen *d*=2.68). In our single-site pilot study, we observed a large increase in the average number of monthly naloxone doses dispensed (from 9.67 doses/month prior to the intervention to 19 doses/month during the intervention). We further do not expect that the control pharmacies will substantially vary in the number of naloxone doses dispensed over time.

We made the decision to conduct this smaller, pilot randomized controlled trial, because although our single-site pilot study produced a sizeable effect on naloxone dispensing, we could not be certain that it was not attributable in part to unmeasured confounding (eg, external trends in naloxone dispensing). In addition, recruiting and providing materials (especially naloxone) to a large sample of pharmacies represents a substantial cost, and so, prior to investing in a more conservative, fully powered, and pragmatic study, we wanted to obtain additional data. Thus, we plan to report all *P* values exactly rather than by threshold and undertake a nuanced interpretation of the findings [45], including a visual inspection and presentation of the trends in intervention and control pharmacies, with the purpose of determining whether a large, randomized study appears to be an appropriate and valuable investment.

Since the pharmacy manager has agreed in principle to participation among all 7 individual pharmacies, we do not intend to apply any inclusion or exclusion criteria but note that individual-level data collection will still be subject to exclusion via declining to participate at the point of informed consent.

### Analysis Plan

#### Primary Outcomes

The number of naloxone doses dispensed will be treated as the overall number of doses, which is the sum of those that are (1) sold, as tracked by the pharmacy’s record system; and (2) dispensed free of cost, as tracked by sequentially numbered doses stored separately in the pharmacy. This value will be obtained retrospectively for the 3 months prior to the intervention and then monthly for 3 months (the duration of the intervention).

#### Secondary Outcomes

As noted previously, this study will collect, describe, and in some cases analyze secondary outcomes to facilitate the interpretation of the study findings. These data and analyses will be treated as exploratory.

We will produce a count of the referral cards distributed by each intervention pharmacy by manual inspection of the individually numbered cards provided to each pharmacy. In addition, using a pretest-posttest methodology, we will collect individual-level data from pharmacy staff members (pharmacists and pharmacy technicians). Measures collected prior to the intervention will include the following (see Multimedia Appendix 1 for a copy of each instrument):

- Sociodemographic information (age, gender, race, ethnicity, and sexual identity);
- Information about current pharmacy practice experience;
- Comfort with multiple harm reduction practices, as used in our prior study to explore harm reduction latent classes [27]; and
- Measures of stigmatizing beliefs about people who use drugs, which combine components of the 2019 Brief Opioid Stigma Scale by Yang et al [46] and work by author BEM’s interdisciplinary group of medication for opioid use disorder patients and providers, which is in review.

The postintervention survey will include the comfort and stigma items (above) as well as additional sets of items (see Multimedia Appendices 2-3):

- Intervention acceptability (4 questions adapted from the Consolidated Framework for Implementation Research [33]);
- A set of questions targeted at implementation optimization validated by Livet et al [47]; and
- Four evaluative items developed by our team to potentially inform future work (eg, “If you could wave a magic wand, how would you improve the PharmNet intervention?”).

#### Statistical Analyses

We will analyze the primary outcome using a generalized linear mixed model with a Poisson distribution to compare change over time of the number of naloxone doses dispensed between study arms. We will include fixed effects for group, time, and their interaction and a random effect for pharmacy ID to account for repeated measures within pharmacy.

We do not anticipate any missingness in the primary outcome because the data are objective measures and can be accessed retrospectively if needed. If missing data are present in the individual-level data (>5%), we will perform multiple imputation by chained equations to assess the degree to which missingness affects the results. If missing data meaningfully affect the findings, we will report the analyses with imputation. If individuals provide a survey at one time point but not another (eg, a pretest without a posttest or vice versa), we will analyze the data as unmatched in our mixed models.

#### Data Management and Monitoring

As noted earlier in this paper, individual-level data from pharmacy staff members will be collected in QualtricsXM, which is a secure platform. Only the research team will have access to the raw data in QualtricsXM. Any identifying information used to match pretest and posttest data will be removed prior to the distribution of the data set for analysis. Based on the opinion of a professional biostatistician, additional data may be redacted prior to public data set release in the specific case that it might allow the identification of an individual staff member.

#### Harms

We do not anticipate that any harms would be accrued to participating pharmacies as a result of participating in this study. Based on our single-site pilot study, we strongly suspect that the burden on staff members will be minimal (since this is a pragmatic study, that is by design). However, pharmacies will always have the option to withdraw from the trial. In the unlikely event that such a thing occurs, we will report the data as they exist and describe the impetus behind the withdrawal in our final write-up. Similarly, we do not anticipate any risks to individual pharmacy staff participants related to completing study questionnaires, which are voluntary. However, we will follow all appropriate IRB procedures in the event of a loss of confidentiality.

#### Data and Code Availability

All data and analytic code associated with this project will be made public alongside the article describing the study results. We will publish an open account of the results regardless of the direction or nature of the findings.

## Results

The not human subjects research designation (12339) was applied by the Indiana University IRB on August 2, 2021. The “exempt” declaration (13956) was granted by the same IRB on April 26, 2022.

## Discussion

### Next Steps

This study is designed to be a pragmatic, pilot randomized controlled trial of the PharmNet intervention. We plan to move forward with preparations for a much larger trial if either 1 of the following 2 cases are true: (1) Hypothesis 1 is upheld and there is evidence of a strong, significant effect of the intervention compared to control; or (2) Hypothesis 1 is not upheld (eg, the primary effect is nonsignificant), but the visual and numeric inspection of differences suggests a reasonable inference that favorable differences might be observed given a fully powered study.

### Limitations

The proposed study is a pragmatic pilot trial, which imposes some limitations on the types of data and nature of information that can be collected, as our goal is to demonstrate real-world effectiveness. In addition, we expect that limitations may be identified during the course of study completion, and they will be identified and described in the results-oriented manuscript to follow the experimental intervention.

## Appendix

Multimedia Appendix 1 PharmNet baseline survey.

Multimedia Appendix 2 PharmNet postintervention survey (existing staff only).

Multimedia Appendix 3 PharmNet postintervention survey (new hires only).

## Abbreviations

IRB: institutional review board
MOU: memorandum of understanding

## Data Availability

Data sharing is not applicable to this article as no data sets were generated or analyzed in preparation of this protocol.
