# Supplementary material for: The PharmNet Harm Reduction Intervention for Community Pharmacies: Protocol for a Pilot Randomized Controlled Trial
Source: JMIR Res Protoc. 2022 Oct 24;11(10):e42373. doi: 10.2196/42373 (PMC9641511; doi:10.2196/42373)
Supplement: Multimedia Appendix 2 [file resprot_v11i10e42373_app2.pdf]

PharmNet Post-Intervention Survey EXISTING STAFF ONLY

Start of Block: Default Question Block

PharmNet - A Harm Reduction Pharmacy Intervention

Thank you for being willing to participate in our second (of two) surveys about PharmNet! Your responses are appreciated and will be used to help advance our understanding of pharmacy harm reduction.

End of Block: Default Question Block

Start of Block: Practice

Page Break

I am *comfortable* with the following pharmacy practices:

	Agree	No opinion	Disagree
Consulting with patients about safe syringe use.	○	○	○
Consulting with patients about safe syringe disposal.	○	○	○
Consulting with patients about safer sex practices.	○	○	○
Consulting with patients about the need for naloxone.	○	○	○
Consulting with patients about their need to reduce opioid misuse.	○	○	○

Page Break

I am *comfortable* with the following pharmacy practices:

	Agree	No opinion	Disagree
Consulting with patients about PrEP for HIV prevention.	○	○	○
Dispensing syringes for non-prescription injection drug use.	○	○	○
Dispensing naloxone for overdose reversal.	○	○	○
Dispensing PrEP for HIV prevention.	○	○	○
Making referrals to community services.	○	○	○

End of Block: Practice

Start of Block: Beliefs

Please indicate your level of agreement with the following statements:

	Strongly agree	Agree	Neither agree nor disagree	Disagree	Strongly disagree
I prefer not to work with patients who use drugs.	○	○	○	○	○
I feel especially compassionate toward patients with opioid use disorder.	○	○	○	○	○
Treating patients with opioid use disorder is a waste of medical dollars.	○	○	○	○	○
I sometimes feel judged by my peers for seeking to serve patients with opioid use disorder.	○	○	○	○	○
I don't want my pharmacy to be known as a supporter of drug users.	○	○	○	○	○

Page Break

Please indicate your level of agreement with the following statements:

	Strongly agree	Agree	Neither agree nor disagree	Disagree	Strongly disagree
Most people believe that a person addicted to opioids is dangerous.	○	○	○	○	○
Most people believe that a person who is addicted to opioids is to blame for their problems.	○	○	○	○	○
I believe that a person who is addicted to opioids cannot be trusted.	○	○	○	○	○
I think that a person who is addicted to opioids is to blame for their problems.	○	○	○	○	○
I believe that patients who are addicted to opioids might cause trouble in my pharmacy.	○	○	○	○	○

End of Block: Beliefs

Start of Block: CFIR

Please indicate your level of agreement with the following statements:

	Strongly agree	Agree	Slightly Agree	Slightly disagree	Disagree	Strongly disagree
I felt prepared to complete my responsibilities with PharmNet after receiving instructions for how to do so.	○	○	○	○	○	○
Our patients asked about naloxone or related services when PharmNet advertising was present (e.g., yard sign, scrolling television ad).	○	○	○	○	○	○
The PharmNet implementation resources (resource cards for patients, reminder post-it notes, access to free naloxone) helped me implement PharmNet.	○	○	○	○	○	○
I felt prepared to implement PharmNet with the amount of coaching that I received.	○	○	○	○	○	○

End of Block: CFIR

Start of Block: Livet

Please indicate your level of agreement with the following statements:

	Strongly agree	Agree	Slightly agree	Slightly disagree	Disagree	Strongly disagree
I believe that PharmNet services are important to deliver at my pharmacy because my patients will benefit.	○	○	○	○	○	○
I support the delivery of PharmNet services in my pharmacy.	○	○	○	○	○	○
PharmNet easily fits within pharmacy flow.	○	○	○	○	○	○
I am not interested in implementing PharmNet.	○	○	○	○	○	○
I am committed to carrying out PharmNet in my pharmacy.	○	○	○	○	○	○

Page Break

Please indicate your level of agreement with the following statements:

	Strongly agree	Agree	Slightly agree	Slightly disagree	Disagree	Strongly disagree
The staff time required to carry out PharmNet is reasonable.	○	○	○	○	○	○
The space needed to carry out PharmNet services is reasonable.	○	○	○	○	○	○
The amount of time required for PharmNet documentation (e.g., naloxone tracking) is reasonable.	○	○	○	○	○	○
The PharmNet instructions and materials are easy to understand and use.	○	○	○	○	○	○
The amount of time required to implement PharmNet is manageable for me.	○	○	○	○	○	○

End of Block: Livet

Start of Block: Block 8

Is PharmNet something that your pharmacy will likely continue to implement?

- ○ No
- ○ Yes
- ○ I don't know (unsure)
- ○ I don't know (outside of my scope of decisionmaking)

Display This Question:

If Is PharmNet something that your pharmacy will likely continue to implement? = Yes

Or Is PharmNet something that your pharmacy will likely continue to implement? = I don't know (unsure)

If you continued with PharmNet, what components would your pharmacy likely continue using?

- ☐ Provide free naloxone on request (if we can get it)
- ☐ Provide community resource cards to patients
- ☐ Advertise about naloxone availability in your pharmacy
- ☐ Other (please identify)

____

Display This Question:

If Is PharmNet something that your pharmacy will likely continue to implement? = No

Please explain (in your opinion) why you do not think your pharmacy will continue to offer PharmNet services.

____

____

____

____

If you could wave a magic wand, how would you improve the PharmNet intervention?

____

____

____

____

____

End of Block: Block 8

Start of Block: Card

Thank you for completing our final survey! We will prepare and send you a $10 digital giftcard to the e-mail with which we invited you to take this survey.

We will never use this e-mail address to contact you except to send you the gift card and activation code.

End of Block: Card
