# Supplementary material for: The PharmNet Harm Reduction Intervention for Community Pharmacies: Protocol for a Pilot Randomized Controlled Trial
Source: JMIR Res Protoc. 2022 Oct 24;11(10):e42373. doi: 10.2196/42373 (PMC9641511; doi:10.2196/42373)
Supplement: Multimedia Appendix 3 [file resprot_v11i10e42373_app3.pdf]

PharmNet Post-Intervention Survey NEW HIRES ONLY

Start of Block: Default Question Block

PharmNet - A Harm Reduction Pharmacy Intervention

You are being asked to participate in a research study. Scientists do research to answer important questions that might help change or improve the way we do things in the future. This consent form will give you information about the study to help you decide whether you want to participate. Please read this form, and ask any questions you have, before agreeing to be in the study.

The purpose of this study is to determine the feasibility and acceptability of implementing PharmNet, a pharmacy harm reduction intervention, in a community pharmacy setting and to determine the impact of PharmNet implementation on pharmacy dispensing of naloxone.

We are asking you if you want to be in this study because you work at as a pharmacy tech or a pharmacist at a community pharmacy. The study is being conducted by Dr. Jon Agley at Indiana University, Bloomington School of Public Health (IRB #13956). It is funded by Indiana University Grand Challenge.

Completing surveys for PharmNet is voluntary. You can choose not to take any or all of the surveys. If you decide to participate, you can change your mind later and leave the survey at any time. You will not be penalized or lose any benefits if you decide not to participate or choose to leave the study later.

This is the second and final survey for PharmNet. Before agreeing to participate, please consider the risks and potential benefits of taking part in this study. You may be uncomfortable while answering the survey questions. While completing the survey, you can skip any questions that make you uncomfortable or that you do not want to answer. We don't think you will have any personal benefits from taking part in this study, but we hope to learn things that will help researchers work better with pharmacies in the future.

You will be paid for participating in this study. Payments will be distributed using digital gift

cards. Completing this survey will be paid at $10.00 USD. There is no cost to participate in the study.

In order to pay you, we will ask you for your e-mail address. We will protect your information and make every effort to keep your personal information confidential, but we cannot guarantee absolute confidentiality. No information which could identify you will be shared in publications about this study.

Your personal information may be shared outside the research study if required by law. We also may need to share your research records with other groups for quality assurance or data analysis. These groups include the Indiana University Institutional Review Board or its designees, and state or federal agencies who may need to access the research records (as allowed by law).

Information collected in this study may be used for other research studies or shared with other researchers for future research. If this happens, information that could identify you, such as your e-mail address, will be removed before any information or specimens are shared. Since identifying information will be removed, we will not ask for your additional consent.

If you have questions about the study or encounter a problem with the research, contact the researcher, Jon Agley, at 812-855-3123 or jagley@indiana.edu. For questions about your rights as a research participant, to discuss problems, complaints, or concerns about a research study, or to obtain information or to offer input, please contact the IU Human Research Protection Program office at 800-696-2949 or at irb@iu.edu.

If you decide to participate in this study, you can change your mind and decide to leave the study at any time in the future. If you decide to withdraw, you you may notify Jon Agley at 812-855-3123 or jagley@indiana.edu to request deletion of your responses. Withdrawal from this study does not pose any risk to you.

○ YES - In consideration of the above, I agree to participate in this research study.

○ NO - I am declining to participate in this research study.

End of Block: Default Question Block

Start of Block: Demographics1

What is your current role at the pharmacy?

○ Pharmacist

○ Pharmacy Technician

○ Other - Please Specify ____

---

*

In what year did you complete your most recent pharmacy-related degree or certification? (*please type 4 digits, like* 2005*)*

____

Page Break

In what year did you begin employment as either a pharmacist or pharmacy technician at your current pharmacy? (*please type 4 digits, like* 2005)

____

In what year did you begin working as either a pharmacist or pharmacy technician at any pharmacy? (that is, we are asking for the year when you first started working in that role, in general) (*please type 4 digits, like* 2005)

____

End of Block: Demographics1

Start of Block: Practice

I am *comfortable* with the following pharmacy practices:

	Agree	No opinion	Disagree
Consulting with patients about safe syringe use.	○	○	○
Consulting with patients about safe syringe disposal.	○	○	○
Consulting with patients about safer sex practices.	○	○	○
Consulting with patients about the need for naloxone.	○	○	○
Consulting with patients about their need to reduce opioid misuse.	○	○	○

Page Break

I am *comfortable* with the following pharmacy practices:

	Agree	No opinion	Disagree
Consulting with patients about PrEP for HIV prevention.	○	○	○
Dispensing syringes for non-prescription injection drug use.	○	○	○
Dispensing naloxone for overdose reversal.	○	○	○
Dispensing PrEP for HIV prevention.	○	○	○
Making referrals to community services.	○	○	○

End of Block: Practice

Start of Block: Beliefs

Please indicate your level of agreement with the following statements:

	Strongly agree	Agree	Neither agree nor disagree	Disagree	Strongly disagree
I prefer not to work with patients who use drugs.	○	○	○	○	○
I feel especially compassionate toward patients with opioid use disorder.	○	○	○	○	○
Treating patients with opioid use disorder is a waste of medical dollars.	○	○	○	○	○
I sometimes feel judged by my peers for seeking to serve patients with opioid use disorder.	○	○	○	○	○
I don't want my pharmacy to be known as a supporter of drug users.	○	○	○	○	○

Page Break

Please indicate your level of agreement with the following statements:

	Strongly agree	Agree	Neither agree nor disagree	Disagree	Strongly disagree
Most people believe that a person addicted to opioids is dangerous.	○	○	○	○	○
Most people believe that a person who is addicted to opioids is to blame for their problems.	○	○	○	○	○
I believe that a person who is addicted to opioids cannot be trusted.	○	○	○	○	○
I think that a person who is addicted to opioids is to blame for their problems.	○	○	○	○	○
I believe that patients who are addicted to opioids might cause trouble in my pharmacy.	○	○	○	○	○

End of Block: Beliefs

Start of Block: CFIR

Please indicate your level of agreement with the following statements:

	Strongly agree	Agree	Slightly Agree	Slightly disagree	Disagree	Strongly disagree
I felt prepared to complete my responsibilities with PharmNet after receiving instructions for how to do so.	○	○	○	○	○	○
Our patients asked about naloxone or related services when PharmNet advertising was present (e.g., yard sign, scrolling television ad).	○	○	○	○	○	○
The PharmNet implementation resources (resource cards for patients, reminder post-it notes, access to free naloxone) helped me implement PharmNet.	○	○	○	○	○	○
I felt prepared to implement PharmNet with the amount of coaching that I received.	○	○	○	○	○	○

End of Block: CFIR

Start of Block: Livet

Please indicate your level of agreement with the following statements:

	Strongly agree	Agree	Slightly agree	Slightly disagree	Disagree	Strongly disagree
I believe that PharmNet services are important to deliver at my pharmacy because my patients will benefit.	○	○	○	○	○	○
I support the delivery of PharmNet services in my pharmacy.	○	○	○	○	○	○
PharmNet easily fits within pharmacy flow.	○	○	○	○	○	○
I am not interested in implementing PharmNet.	○	○	○	○	○	○
I am committed to carrying out PharmNet in my pharmacy.	○	○	○	○	○	○

Page Break

Please indicate your level of agreement with the following statements:

	Strongly agree	Agree	Slightly agree	Slightly disagree	Disagree	Strongly disagree
The staff time required to carry out PharmNet is reasonable.	○	○	○	○	○	○
The space needed to carry out PharmNet services is reasonable.	○	○	○	○	○	○
The amount of time required for PharmNet documentation (e.g., naloxone tracking) is reasonable.	○	○	○	○	○	○
The PharmNet instructions and materials are easy to understand and use.	○	○	○	○	○	○
The amount of time required to implement PharmNet is manageable for me.	○	○	○	○	○	○

End of Block: Livet

Start of Block: Post

Is PharmNet something that your pharmacy will likely continue to implement?

- ◯ No
- ◯ Yes
- ◯ I don't know (unsure)
- ◯ I don't know (outside of my scope of decisionmaking)

Display This Question:

If Is PharmNet something that your pharmacy will likely continue to implement? = Yes

Or Is PharmNet something that your pharmacy will likely continue to implement? = I don't know (unsure)

If you continued with PharmNet, what components would your pharmacy likely continue using?

- ▢ Provide free naloxone on request (if we can get it)
- ▢ Provide community resource cards to patients
- ▢ Advertise about naloxone availability in your pharmacy
- ▢ Other (please identify) ____

Page Break

Display This Question:

If Is PharmNet something that your pharmacy will likely continue to implement? = No

Please explain (in your opinion) why you do not think your pharmacy will continue to offer PharmNet services.

____

____

____

____

____

If you could wave a magic wand, how would you improve the PharmNet intervention?

____

____

____

____

____

End of Block: Post

Start of Block: Demographics2

*

How old are you (in *years*)?

____

Which of the following best describes you?

- ○ Woman (cisgender woman, transgender woman, and female-identified individuals)
- ○ Man (cisgender man, transgender man, and male-identified individuals)
- ○ Prefer not to answer
- ○ Prefer to self-describe ____

Page Break

What is your race?

- ◯ American Indian or Alaska Native
- ◯ Asian
- ◯ Black or African American
- ◯ White or Caucasian
- ◯ Native Hawaiian or Other Pacific Islander
- ◯ Prefer not to answer
- ◯ Prefer to self-describe ____________________

What is your ethnicity?

- ◯ Hispanic or Latino/a
- ◯ Not Hispanic or Latino/a
- ◯ Prefer not to answer
- ◯ Prefer to self-describe ____________________

How would you describe your sexual identity?

- ○ Heterosexual/Straight
- ○ Homosexual/Gay/Lesbian
- ○ Bisexual
- ○ Other
- ○ Prefer not to answer
- ○ Prefer to self-describe ____

End of Block: Demographics2

Start of Block: Card

*

Thank you for completing our final survey! If you would like to receive a $10 digital giftcard in your e-mail, please type it in the text box below.

We will never use this e-mail address to contact you except to send you the gift card and activation code.

____

End of Block: Card
